# *Monolluma quadrangula* Protects against Oxidative Stress and Modulates LDL Receptor and Fatty Acid Synthase Gene Expression in Hypercholesterolemic Rats

**DOI:** 10.1155/2018/3914384

**Published:** 2018-09-30

**Authors:** May N. Bin-Jumah

**Affiliations:** Department of Biology, College of Science, Princess Nourah bint Abdulrahman University, Riyadh, Saudi Arabia

## Abstract

Hypercholesterolemia is a metabolic disorder associated with oxidative stress. The present study investigated the protective effect of *Monolluma quadrangula* extract on hypercholesterolemia-induced oxidative stress in the liver and heart of high-cholesterol-diet- (HCD-) fed rats. The experimental animals received HCD for 10 weeks and were concurrently treated with 300 or 600 mg/kg *M. quadrangula* extract. HCD-fed rats showed a significant increase in serum triglycerides, total cholesterol, LDL-cholesterol, vLDL-cholesterol, and cardiovascular risk indices along with decreased HDL-cholesterol and antiatherogenic index. The *M. quadrangula* extract significantly improved dyslipidemia and atherogenesis in HCD-fed rats. HCD induced a significant increase in serum transaminases, creatine kinase-MB, and proinflammatory cytokines. In addition, HDC induced a significant increase in hepatic and cardiac lipid peroxidation and decreased antioxidant enzymes. Treatment with the *M. quadrangula* extract significantly alleviated liver and heart function markers, decreased proinflammatory cytokines and lipid peroxidation, and enhanced the antioxidant defenses. Also, the *M. quadrangula* extract significantly reduced the expression of fatty acid synthase (FAS) and increased the expression of LDL receptor in the liver of HCD-fed rats. In conclusion, the *M. quadrangula* extract has a potent antihyperlipidemic and cholesterol-lowering effect on HCD-fed rats. The beneficial effects of the *M. quadrangula* extract were mediated through the increased antioxidant defenses, decreased inflammation and lipid peroxidation, and modulated hepatic FAS and LDL receptor gene expression.

## 1. Introduction

Hyperlipidemia is a lipid metabolism disorder associated with the development of atherosclerosis and cardiovascular disease [1, 2]. Excessive consumption of foods containing high amounts of saturated fats and cholesterol is the major risk factor of hyperlipidemia [3]. Studies have reported that hyperlipidemia, particularly hypercholesterolemia, leads to atherosclerosis which is a chronic inflammatory status initiated by subendothelial retention and oxidation of low-density lipoprotein (LDL) cholesterol [4].

Dyslipidemia/hypercholesterolemia leads to increased accumulation of lipids in the liver, hence reducing its ability to lower blood lipids [2]. Studies have reported the significant role of hypercholesterolemia in inducing oxidative stress [2, 5, 6]. Increased production of free radicals and decreased enzymatic and nonenzymatic antioxidants are the main features of oxidative stress [7]. Cholesterol accumulation in endothelial cells, hepatocytes, leukocytes, erythrocytes, and platelets provokes the production of reactive oxygen species (ROS) and reduces antioxidant defenses [7, 8]. This can lead to redox imbalance, oxidative stress, and metabolic alterations [9, 10]. Therefore, agents that combine lipid-lowering and antioxidant potentials can prevent the negative impact of cholesterol on the liver, heart, and other body tissues.

Ethnopharmacological data has been the base for the discovery of natural drugs from medicinal plants [11]. *Monolluma quadrangula* (Forssk.) Plowes belongs to family *Apocynaceae* and is also known as *Caralluma quadrangula* [11]. Plants of the *Caralluma* spp. have shown different beneficial effects on the treatment of skin rashes, diabetes, snake bites, inflammation, and cancer [12–16]. *M. quadrangula* is a succulent plant that has been used traditionally as an appetite suppressant and as diabetes and peptic ulcer treatment. Recently, the *M. quadrangula* hydroethanolic extract has been reported to protect against ethanol-induced gastric ulcers and to attenuate oxidative stress in rats [17]. The mechanism of the antihypercholesterolemic effect of *M. quadrangula* has not been previously investigated. Therefore, we carried out this investigation to study the antihypercholesterolemic effect of *M. quadrangula* and its protective effect against hypercholesterolemia-induced oxidative stress in the liver and heart of rats. In addition, we studied the effect of *M. quadrangula* on the gene expression levels of hepatic LDL receptor (LDLR) and fatty acid synthase (FAS) in hypercholesterolemic rats.

## 2. Materials and Methods

### 2.1. Collection and Preparation of the *M. quadrangula* Extract

*M. quadrangula* was collected from Abha-Al-Taif road (Kingdom of Saudi Arabia) during the period from September to November 2017. The plant samples were identified and authenticated by an expert taxonomist. The collected samples were air-dried in shade, and the hydroethanolic extract was prepared as previously described [17]. Briefly, the dried *M. quadrangula* samples were ground into a fine powder and soaked in ethanol/water (1 : 1 vol/vol) for 24 hr. The mixture was filtered, and the solvent was evaporated in a rotary evaporator at temperature not exceeding 45°C. The dried extract was kept frozen at −20°C until it will be used in the animal experiments.

### 2.2. Experimental Animals and Treatments

Male albino Wistar rats, weighing 160–180 g, were obtained from the animal house of King Saud University (Saudi Arabia). The rats were housed in standard well-aerated cages (4 rats/cage) in controlled 12 hr dark/light cycles. The animals were provided a standard diet of known composition with free access to water. All animal procedures were approved by the ethical committee at Princess Nourah bint Abdulrahman University (Riyadh, Saudi Arabia).

After a 10-day acclimatization period, the rats were allocated into four groups, each comprising 8 rats. Group I included control rats supplied with a normal diet for 10 weeks and a daily oral dose of distilled water. Groups II–IV included rats fed with a hypercholesterolemic diet for 10 weeks. The hypercholesterolemic diet consisted of a normal diet supplemented with 2% cholesterol. Group II received distilled water daily via oral gavage for 10 weeks. Groups III and IV received 300 and 600 mg/kg body weight *M. quadrangula*, respectively, dissolved in distilled water via oral gavage daily for 10 weeks. The doses of *M. quadrangula* were selected based on the study of Ibrahim et al. [17] who showed that rats receiving up to 5 g/kg body weight *M. quadrangula* extract showed no signs of hepatotoxicity or nephrotoxicity and no morbidity or mortality.

### 2.3. Sample Collection and Preparation

At the end of the experiment, both the control and treated groups were fasted overnight and sacrificed under anesthesia. Blood samples were quickly collected in 5 ml sterilized glass tubes, were left to coagulate, and then centrifuged to separate serum. Immediately, the rats were dissected, and the liver and heart were excised, washed in cold phosphate-buffered saline (PBS), and weighed. Samples from the liver and heart were homogenized in cold 0.1 M phosphate buffer (pH 7.4) using a polytron homogenizer. The homogenate was centrifuged at 8000 rpm in a cooling centrifuge, and the clear supernatant was collected and stored at −80°C for biochemical analyses.

### 2.4. Assay of Liver and Heart Function Enzymes

Alanine transaminase (ALT) and aspartate transaminase (AST) were determined in serum of rats using commercial kits (Spinreact, Spain) based on the method of Reitman-Frankel [25]. Serum creatine kinase-MB (CK-MB) was determined using the commercial ELISA kit (EIAab, China) [18].

### 2.5. Determination of Serum Lipids and Cardiovascular Risk Indices

The levels of triglycerides [19], total cholesterol [20], and HDL-cholesterol [21] were determined in the serum of rats using a commercially available reagent kit supplied by Accurex (Mumbai, India). vLDL- and LDL-cholesterol levels were calculated using the following formulas: vLDL-cholesterol = triglycerides/5 and LDL-cholesterol = total cholesterol − (HDL-cholesterol + vLDL-cholesterol).

Cardiovascular risk indices [22] and antiatherogenic index (AAI) [23] were calculated as follows: cardiovascular risk index 1 = total cholesterol/HDL-cholesterol, cardiovascular risk index 2 = LDL-cholesterol/HDL-cholesterol, and AAI = (HDL-cholesterol × 100)/(total cholesterol − HDL-cholesterol).

### 2.6. Assay of Serum Cytokines and C-reactive Protein (CRP)

Serum levels of tumor necrosis factor alpha (TNF-*α*), interleukin-6 (IL-6), and CRP were determined using commercially available ELISA kits (Merck Millipore, USA) according to the provided instructions.

### 2.7. Assay of Lipid Peroxidation and Antioxidants

The levels of lipid peroxidation, reduced glutathione (GSH), superoxide dismutase (SOD), and catalase (CAT) were determined in the homogenates of the liver and heart following the instructions of the assay kits purchased from OxiSelect (USA).

### 2.8. Gene Expression Assay of LDL Receptor (LDLR) and Fatty Acid Synthase (FAS)

Quantitative polymerase chain reaction (qPCR) was used to analyze the mRNA expression levels of LDLR and FAS in the liver of control and treated rats. Total RNA was extracted using the RNA Mini kit (Bioline, USA) according to the manufacturer's protocol. RNA was quantified using Nanodrop 8000 (Thermo Scientific, USA), and samples with a 260/280 absorbance ratio of 1.8–2.0 were reverse-transcribed into cDNA using a reverse transcription kit (Invitrogen, USA). cDNA amplification was carried out using SYBR green (Invitrogen, USA) and the following primer pairs (Metabion International AG, Germany): LDLR (F: 5′-CAGCTCTGTGTGAACCTGGA-3′ and R: 5′-TTCTTCAGGTTGGGGATCAG-3′), FAF (F: 5′-CTGGACTCGCTCATGGGTG-3′ and R: 5′-CATTTCCTGAAGCTTCCGCAG-3′), and GAPDH (F: 5′-AACTTTGGCATCGTGGAAGG-3′ and R: 5′-TACATTGGGGGTAGGAACAC-3′). The results were analyzed using the 2^−ΔΔCt^ method of analysis as described by Livak and Schmittgen [24].

### 2.9. Statistical Analysis

All statistical comparisons were made by means of the one-way ANOVA test followed by Tukey's test on GraphPad Prism (GraphPad Software, CA, USA). Results were presented as mean ± standard error (SEM), and a *P* value less than 0.05 was considered significant.

## 3. Results

### 3.1. Effect of the *M. quadrangula* Extract on Serum Lipids, Cardiovascular Indices, and Antiatherogenic Index

**Figure 1:**
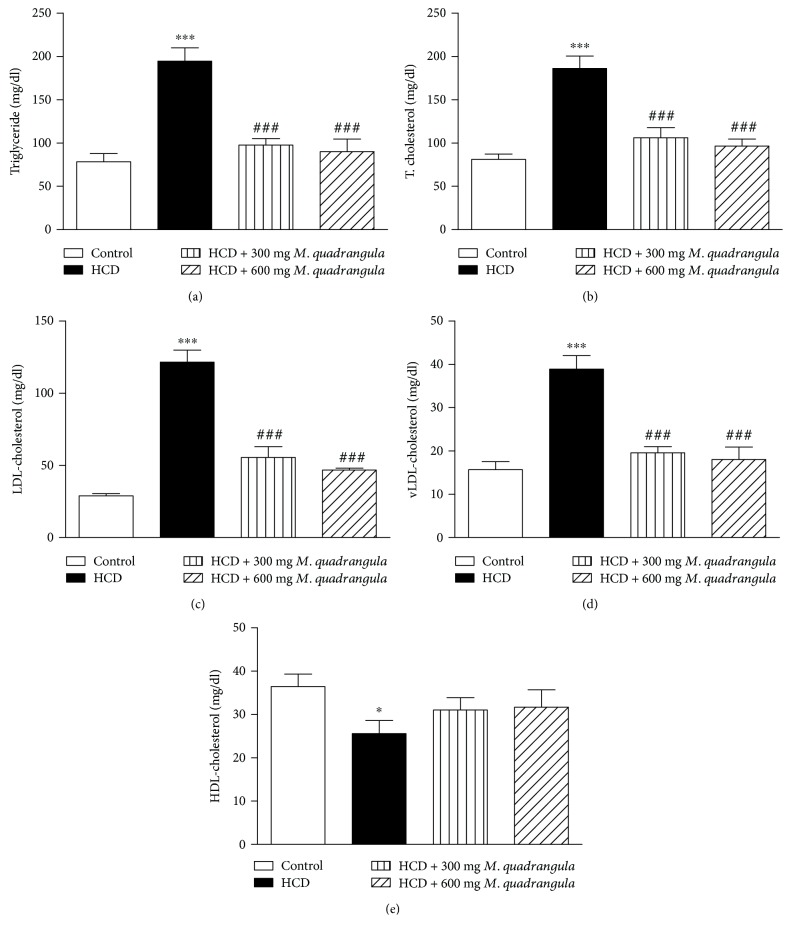
Effect of the *M. quadrangula* extract on serum (a) triglycerides, (b) total cholesterol, (c) LDL-cholesterol, (d) vLDL-cholesterol, and (e) HDL-cholesterol of high-cholesterol-diet-fed rats. Data are mean ± SEM. The number of animals in each group is eight. ^∗^*P* < 0.05 and ^∗∗∗^*P* < 0.001 compared to control. ^###^*P* < 0.001 compared to high-cholesterol diet.

In the present study, the effect of the *M. quadrangula* extract on the lipid profile in HCD-supplemented rats was evaluated as shown in Figure 1. When compared with the control group, HCD-induced rats showed a significant increase in serum levels of triglycerides (*P* < 0.001), total cholesterol (*P* < 0.001), LDL-cholesterol (*P* < 0.001), and vLDL-cholesterol (*P* < 0.001). On the other hand, HCD-induced rats showed a significant (*P* < 0.05) decrease in serum levels of HDL-cholesterol (Figure 1). HCD-induced rats treated with 300 and 600 mg/kg *M. quadrangula* extract showed a significant (*P* < 0.001) alleviation in serum levels of triglycerides, total cholesterol, LDL-cholesterol, and vLDL-cholesterol, while this treatment had a nonsignificant effect on serum HDL-cholesterol levels.

**Figure 2:**
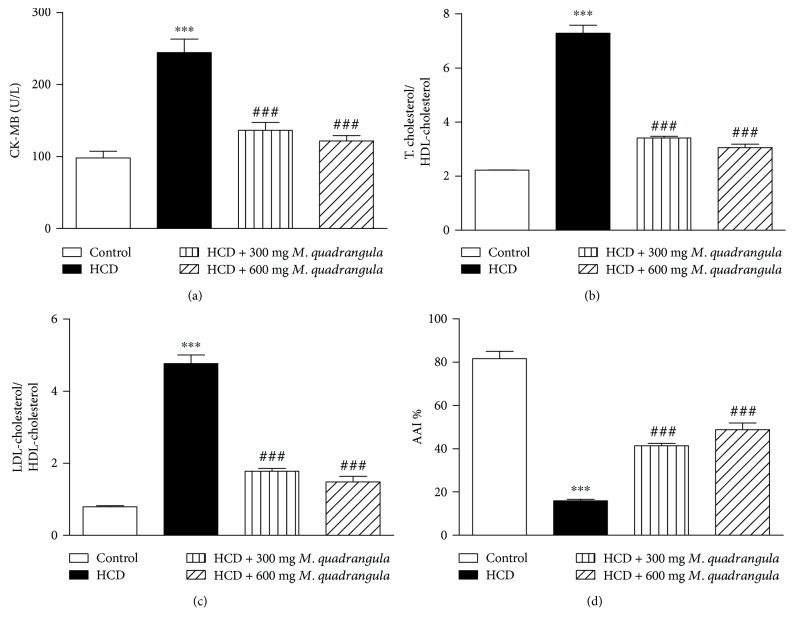
Effect of the *M. quadrangula* extract on (a) serum creatine kinase-MB, (b, c) cardiovascular risk indices, and (d) antiatherogenic index of high-cholesterol-diet-fed rats. Data are mean ± SEM. The number of animals in each group is eight. ^∗∗∗^*P* < 0.001 compared to control. ^###^*P* < 0.001 compared to high-cholesterol diet.

HCD induced cardiac injury as shown by the significant increase in serum CK-MB level (*P* < 0.001) when compared with the control rats (Figure 2(a)). Similarly, HCD-induced rats showed a significant increase in total cholesterol/HDL-cholesterol (Figure 2(b)) and LDL-cholesterol/HDL-cholesterol ratios (Figure 2(c)). On the other hand, HCD-induced rats showed significantly decreased AAI (Figure 2(d)). HCD-induced rats treated with 300 and 600 mg/kg *M. quadrangula* extract showed a significant (*P* < 0.001) alleviation in serum levels of CK-MB and total cholesterol/HDL-cholesterol (Figure 2(b)) and LDL-cholesterol/HDL-cholesterol ratios (Figure 2(c)), and the AAI (Figure 2(d)).

### 3.2. Effect of the *M. quadrangula* Extract on Liver Function Indices

**Figure 3:**
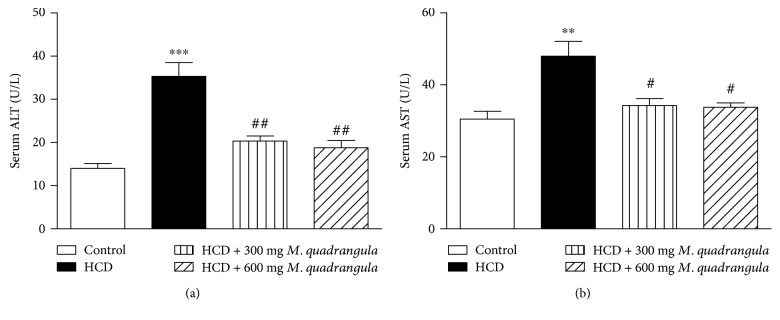
Effect of the *M. quadrangula* extract on serum (a) ALT and (b) AST in high-cholesterol-diet-fed rats. Data are mean ± SEM. The number of animals in each group is eight. ^∗∗^*P* < 0.01 and ^∗∗∗^*P* < 0.001 compared to control. ^#^*P* < 0.05 and ^##^*P* < 0.01 compared to high-cholesterol diet.

Rats receiving HCD for 10 weeks showed significantly increased ALT (*P* < 0.001) and AST (*P* < 0.01) levels in serum (Figure 3). Rats supplemented with the HCD and concurrently treated with 300 and 600 mg/kg *M. quadrangula* extract showed significantly alleviated serum ALT (*P* < 0.01) and AST (*P* < 0.05) levels as represented in Figure 3.

### 3.3. Effect of the *M. quadrangula* Extract on Serum Cytokines and CRP Levels

**Figure 4:**
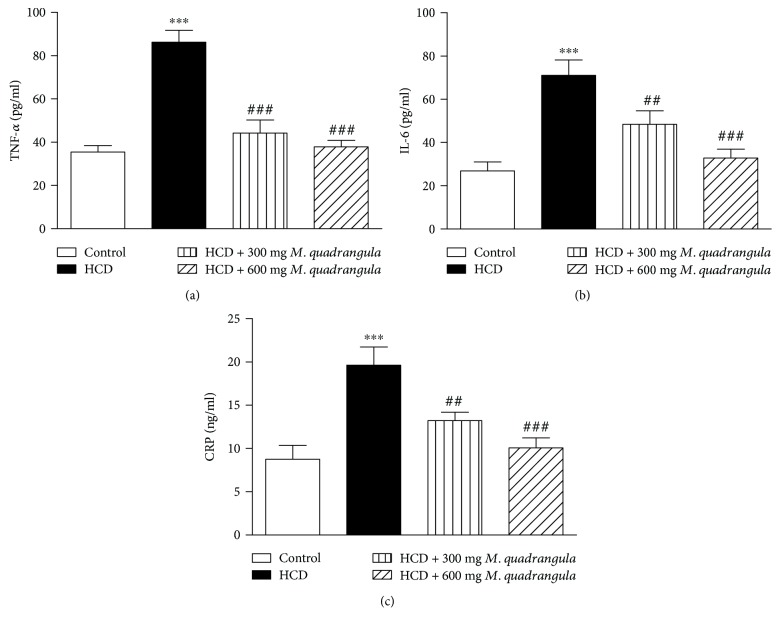
Effect of the *M. quadrangula* extract on serum (a) tumor necrosis factor alpha (b) interleukin-6 and (c) C-reactive protein of high-cholesterol-diet-fed rats. Data are mean ± SEM. The number of animals in each group is eight. ^∗∗∗^*P* < 0.001 compared to control. ^##^*P* < 0.01 and ^###^*P* < 0.001 compared to high-cholesterol diet.

Rats receiving HCD for 10 weeks showed a significant increase in serum TNF-*α* (*P* < 0.001), IL-6 (*P* < 0.001), and CRP (*P* < 0.001) when compared to control rats (Figure 4). Rats supplemented with the HCD and treated with 300 and 600 mg/kg *M. quadrangula* extract showed significantly alleviated serum levels of TNF-*α*, IL-6, and CRP.

### 3.4. Effect of the *M. quadrangula* Extract on Lipid Peroxidation and Antioxidants in the Liver and Heart of HCD-Induced Rats

**Figure 5:**
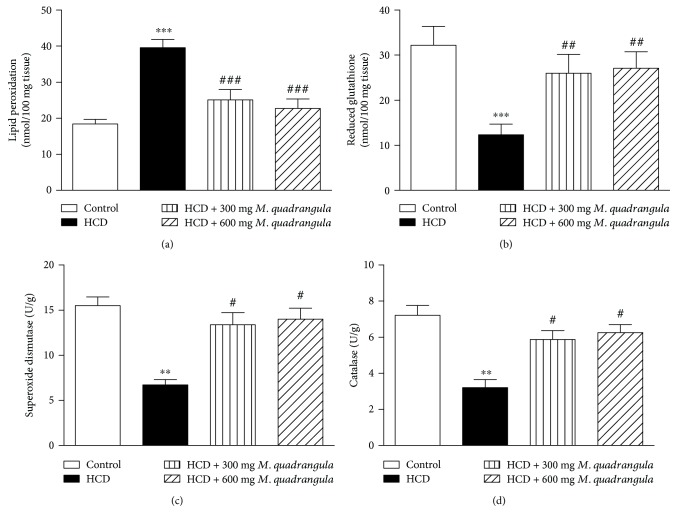
Effect of the *M. quadrangula* extract on (a) lipid peroxidation, (b) reduced glutathione, (c) superoxide dismutase, and (d) catalase in the liver of high-cholesterol-diet-fed rats. Data are mean ± SEM. The number of animals in each group is eight. ^∗∗^*P* < 0.01 and ^∗∗∗^*P* < 0.001 compared to control. ^#^*P* < 0.05, ^##^*P* < 0.01, and ^###^*P* < 0.001 compared to high-cholesterol diet.

**Figure 6:**
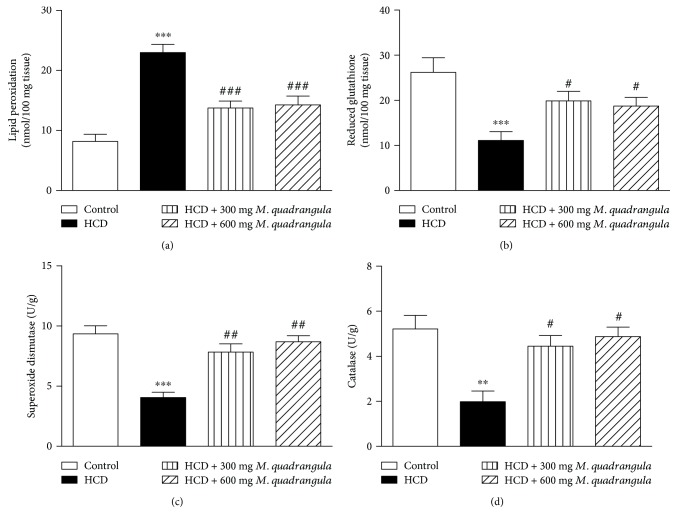
Effect of the *M. quadrangula* extract on (a) lipid peroxidation, (b) reduced glutathione, (c) superoxide dismutase, and (d) catalase in the heart of high-cholesterol-diet-fed rats. Data are mean ± SEM. The number of animals in each group is eight. ^∗∗^*P* < 0.01 and ^∗∗∗^*P* < 0.001 compared to control. ^#^*P* < 0.05, ^##^*P* < 0.01, and ^###^*P* < 0.001 compared to high-cholesterol diet.

Lipid peroxidation level was significantly (*P* < 0.001) increased in the liver (Figure 5(a)) and heart (Figure 6(a)) of rats supplemented with HCD for 10 weeks. On the other hand, levels of GSH in the liver (Figure 5(b)) and heart (Figure 6(b)) of HCD-induced rats were significantly (*P* < 0.001) decreased. The antioxidants SOD and CAT showed to be significantly decreased in the liver (Figures 5(c) and 5(d)) and heart (Figures 6(c) and 6(d)) of HCD-induced rats. Rats supplemented with the HCD and treated with 300 and 600 mg/kg *M. quadrangula* extract showed a significant decrease in liver and heart lipid peroxidation levels and a significant alleviation in the antioxidants GSH, SOD, and CAT.

### 3.5. Effect of the *M. quadrangula* Extract on Gene Expression Levels of LDLR and FAS in the Liver of HCD-Induced Rats

**Figure 7:**
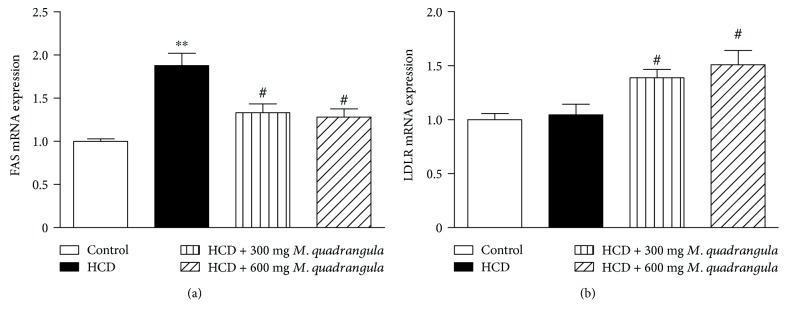
Effect of the *M. quadrangula* extract on gene expression levels of (a) fatty acid synthase and (b) LDL receptor in the liver of high-cholesterol-diet-fed rats. Data are mean ± SEM. The number of animals in each group is eight. ^∗∗^*P* < 0.01 compared to control. ^#^*P* < 0.05 compared to high-cholesterol diet.

FAS mRNA expression analysis in the present study revealed a significant (*P* < 0.01) increase in the liver of HCD-induced rats when compared with the control rats (Figure 7(a)). On the other hand, HCD-induced rats showed a nonsignificant (*P* > 0.05) change in LDLR mRNA expression as represented in Figure 7(b). Treatment of the HCD-induced rats with 300 and 600 mg/kg *M. quadrangula* extract significantly decreased the expression of FAS (*P* < 0.05) and increased the expression of LDLR (*P* < 0.05) in the liver as depicted in Figures 7(a) and 7(b), respectively.

## 4. Discussion

Hyperlipidemia is a lipid metabolism disorder associated with the etiopathogenesis of different diseases, including atherosclerosis, metabolic syndrome, hypertension, renal injury, and cardiovascular disease [18, 25–28]. Hypercholesterolemia has been reported to be implicated in protein glycation, oxidative modification of LDL, and lipid peroxidation [29]. In the present study, we studied the protective effect of the *M. quadrangula* extract against hypercholesterolemia-induced oxidative stress in the liver and heart of rats. In addition, we evaluated the effect of the *M. quadrangula* extract on the lipid profile and the gene expression of LDLR and FAS in the liver of hypercholesterolemic rats.

Rats receiving HCD for 10 weeks exhibited dyslipidemia and hypercholesterolemia as shown by the significant increase in serum total cholesterol, triglyceride, LDL-cholesterol, and vLDL-cholesterol levels. These results were in agreement with different studies showing developed hypercholesterolemia in HCD-fed rats [2, 5, 6]. In addition, HCD-induced rats exhibited a significant decrease in serum levels of HDL-cholesterol. Accordingly, previous reports have shown that elevated total cholesterol, LDL-cholesterol, and vLDL-cholesterol and decreased HDL-cholesterol are the common features of dyslipidemia/hypercholesterolemia irrespective of the etiology [2, 18, 30]. Dyslipidemia can also lead to increased hepatic accumulation of lipids which may reduce the ability of the liver to lower the levels of these lipid components [2]. Increased hepatic lipid accumulation in cases of hypercholesterolemia leads to hepatic cell injury [2]. In the present investigation, HCD-induced rats showed a significant increase in serum levels of ALT and AST, hence leading to liver injury. Interestingly, HCD-induced rats treated with the *M. quadrangula* extract showed a significant improvement in serum lipids and transaminases. These results point to the potent antihypercholesterolemic and hepatoprotective effects of the *M. quadrangula* extract.

Dyslipidemia/hypercholesterolemia can lead to atherosclerosis and cardiovascular disease which represent a major cause of death. Therefore, lowering blood lipids might help counteract the bad impact of hyperlipidemia on the heart. In the present study, HCD-induced rats showed different cardiovascular effects evidenced by the increased values of cardiovascular risk indices and decreased AAI. These results were supported by the increased serum levels of the heart function marker CK-MB. In contrast, HCD-induced rats treated with the *M. quadrangula* extract showed significantly improved serum CK-MB levels, cardiovascular indices, and AAI. These findings are a direct result the improved lipid profile. Hence, the antihyperlipidemic effect of the *M. quadrangula* extract represents the mechanism behind its cardioprotective efficacy.

Oxidative stress has been reported to increase under hypercholesterolemic conditions [2, 5, 6]. In addition, oxidative stress has been suggested to be the mechanism through which hypercholesterolemia induces tissue damage [3, 31]. In the present study, HCD-induced hypercholesterolemic rats showed increased levels of lipid peroxidation and decreased antioxidant defenses in the heart and liver of rats as previously reported [2, 5, 6]. Increased lipid peroxidation and declined antioxidant defenses induced by hyperlipidemia can provoke oxidative stress and lead to injury and cell death. Accordingly, previous studies have demonstrated that obesity/hyperlipidemia is an independent risk factor for increased lipid peroxidation, declined cytoprotective defenses, and cell death [32, 33]. HCD-fed rats treated with the *M. quadrangula* extract showed a significant decrease in lipid peroxidation levels and an increase in GSH, SOD, and CAT in the heart and liver. These findings could be attributed to the improved lipid profile as well as to the antioxidant potential of the *M. quadrangula* extract. In agreement with our findings, Ibrahim et al. [17] have recently reported the antioxidant effect of the *M. quadrangula* extract on a rat model of peptic ulcer. Pretreatment with the *M. quadrangula* extract decreased lipid peroxidation and increased the antioxidant enzymes SOD and CAT in the stomach of a rat model of ethanol-induced gastric ulcer [17].

In addition to induction of oxidative stress, hypercholesterolemia in the present study was associated with increased serum proinflammatory cytokines and CRP. Increased proinflammatory cytokines could be a direct result of hypercholesterolemia-induced production of reactive oxygen species (ROS). ROS are well known to activate nuclear factor-kappa B (NF-*κ*B) which elicits the expression of proinflammatory cytokines, including TNF-*α* and IL-6. These cytokines can induce further production of ROS and hence more cell and tissue damage. Moreover, oxidative modification of LDL particles can induce the expression of adhesive molecules and lead to the secretion of cytokines [4]. The *M. quadrangula* extract significantly decreased serum proinflammatory cytokines in HCD-induced rats. This anti-inflammatory potential of the *M. quadrangula* extract could be a result of its lipid-lowering and antioxidant potential.

The observed lipid-lowering effect of the *M. quadrangula* extract in this study could be exerted, at least in part, through its ability to decrease the gene expression of FAS and increase LDLR expression in the liver of rats. FAS is one of the fatty acid-synthesizing enzymes, and multiple studies have reported its increased expression in the liver of rodents fed with a high-fat or high-cholesterol diet [2, 34]. In the liver, LDLR is primarily responsible for the absorption of circulating cholesterol through LDLR-mediated endocytosis. The absorbed cholesterol via LDLR is then metabolized within the hepatocytes [31, 35]. HCD-induced rats in the present study showed a significant increase in the gene expression of FAS while LDLR has not been affected as recently reported by Lee et al. [2]. In our investigation, we showed for the first time that the *M. quadrangula* extract exerts its lipid-lowering effect through decreasing the expression of hepatic FAS while increasing the expression of LDLR. These beneficial effects of the *M. quadrangula* extract on HCD-fed rats could be linked to its active constituents. Studies have reported the rich content of steroidal and pregnane glycosides, sterols, and flavonoids of *Caralluma* spp. [36, 37]. Flavonoids have been reported to exert multiple effects such as antioxidant, anti-inflammatory, anticarcinogenic, antihyperlipidemic, hepatoprotective, and antidiabetic effects [38–40].

In conclusion, the *M. quadrangula* extract has a potent antihyperlipidemic and cholesterol-lowering effect on HCD-fed rats. The *M. quadrangula* extract significantly decreased serum lipids, proinflammatory cytokines, and heart and liver lipid peroxidation. In addition, the *M. quadrangula* extract increased the antioxidant defenses in the liver and heart of HCD-fed rats. The lipid-lowering effect of the *M. quadrangula* extract was mediated partially through the decreased expression of hepatic FAS and increased expression of LDLR. However, further studies to determine the exact lipid-lowering mechanism of the *M. quadrangula* extract are recommended.

## Data Availability

The data used to support the findings of this study are available from the corresponding author upon request.
